# Identification of In-Chain-Functionalized Compounds and Methyl-Branched Alkanes in Cuticular Waxes of *Triticum aestivum* cv. Bethlehem

**DOI:** 10.1371/journal.pone.0165827

**Published:** 2016-11-07

**Authors:** Radu C. Racovita, Reinhard Jetter

**Affiliations:** 1 Department of Chemistry, The University of British Columbia, Vancouver, BC, V6T 1Z1, Canada; 2 Department of Botany, The University of British Columbia, Vancouver, BC, V6T 1Z4, Canada; Agriculture and Agri-Food Canada, CANADA

## Abstract

In this work, cuticular waxes from flag leaf blades and peduncles of *Triticum aestivum* cv. Bethlehem were investigated in search for novel wax compounds. Seven wax compound classes were detected that had previously not been reported, and their structures were elucidated using gas chromatography-mass spectrometry of various derivatives. Six of the classes were identified as series of homologs differing by two methylene units, while the seventh was a homologous series with homologs with single methylene unit differences. In the waxes of flag leaf blades, secondary alcohols (predominantly C_27_ and C_33_), primary/secondary diols (predominantly C_28_) and esters of primary/secondary diols (predominantly C_50_, combining C_28_ diol with C_22_ acid) were found, all sharing similar secondary hydroxyl group positions at and around C-12 or ω-12. 7- and 8-hydroxy-2-alkanol esters (predominantly C_35_), 7- and 8-oxo-2-alkanol esters (predominantly C_35_), and 4-alkylbutan-4-olides (predominantly C_28_) were found both in flag leaf and peduncle wax mixtures. Finally, a series of even- and odd-numbered alkane homologs was identified in both leaf and peduncle waxes, with an internal methyl branch preferentially on C-11 and C-13 of homologs with even total carbon number and on C-12 of odd-numbered homologs. Biosynthetic pathways are suggested for all compounds, based on common structural features and matching chain length profiles with other wheat wax compound classes.

## Introduction

Most above-ground organs of land plants are covered by a hydrophobic coating, known as the cuticle, which is sealing them against uncontrolled loss of water. The cuticle is composed of the polyester cutin and cuticular wax that can easily be extracted with organic solvents [1]. Cutin consists of saturated and unsaturated long-chain (LC, C_16_ and C_18_) hydroxy or epoxy fatty acids, linked via ester bonds either directly between fatty acids or via glycerol [2,3]. Cuticular waxes are typically very-long-chain (VLC, >C_20_) saturated aliphatic compounds, bearing no functionality or only one functional group at one end of the carbon chain (hence a primary functionality). Most commonly encountered are homologous series of even-numbered fatty acids, primary alcohols, alkyl esters and aldehydes, as well as odd-numbered alkanes, secondary alcohols and ketones [1,4]. In the wax mixtures of some plant species, alicyclics (*e*.*g*., triterpenoids) and aromatics (*e*.*g*., alkylresorcinols) can also be quite abundant [5–9].

The characteristic mixtures of the ubiquitous aliphatic wax constituents (with chain-end functionalities) result from biosynthetic pathways that are fairly well understood, mainly due to extensive studies in the model species *Arabidopsis thaliana* [10,11]. Biosynthesis of the ubiquitous wax compounds begins in the epidermal plastids, where the fatty acid synthases (FASs) elongate acetyl-CoA into LC fatty acids. These are then transferred to the endoplasmic reticulum (ER), where fatty acid elongase (FAE) complexes extend their chain length two carbons at a time to VLC acyls. The resulting VLC fatty acyl-CoAs can then be further processed into final wax compounds, by (1) reduction to primary alcohols which may be esterified with (V)LC fatty acids; or (2) partial reduction to aldehydes, their subsequent decarbonylation to alkanes, and, in Arabidopsis and likely also some other species, hydroxylation of the alkanes to secondary alcohols and ketones.

While the Arabidopsis model has proved invaluable for our understanding of cuticular wax biosynthesis, much can be learned from studying the diversity of wax structures in other plant species as well. Thus, numerous novel wax compounds were discovered recently in various plant species, suggesting divergence from the pathways found in Arabidopsis. Among these new structures, many have multiple functional groups, combining chain-end and in-chain functionalities (primary and secondary functional groups, respectively). Thus, they include diols [12–15], hydroxyaldehydes [16,17], ketols [14], ketoaldehydes [18], hydroxyacids [19], hydroxyesters [19,20] and ketoesters [18]. In many cases, the nature of the functional groups, their relative positions and the chain length profiles within respective homologous series could be used to infer the biosynthetic origin of functional groups or entire molecules. The work herein is an extension of these studies, with the objective of seeking, identifying and quantifying wax compounds with in-chain functional groups to further our understanding of wax biosynthesis beyond the well-known constituents of the Arabidopsis wax mixture.

Bread wheat (*Triticum aestivum*) is rapidly becoming a new model species for wax biosynthesis studies [21–25], due to its importance as a major staple crop world-wide and its susceptibility to drought, combined with the recognized role of cuticular waxes in conferring drought resistance in this species. Diverse reports showed that the waxes of various wheat cultivars all contain typical wax components with chain-end functionalities, together with β-diketones with two carbonyl groups near the middle of the hydrocarbon chain [22,26–30]. The β-diketones have characteristic 1,3-geometry of functional groups, which are positioned largely on even-numbered carbon atoms, for example in the very abundant compound hentriacontane-14,16-dione. Based on their isomer composition, the β-diketones have long been recognized as polyketides, and as such are known to be formed on biosynthetic pathways distinct from those of Arabidopsis [21,31–34]. Very similar aliphatic polyketides occur in the waxes of wheat, barley and many other Poaceae, but also of Eucalypts, Ericaceae and diverse other angiosperms [35–39].

In a previous analysis of flag leaf blade and peduncle waxes of *T*. *aestivum* cv. Bethlehem by gas chromatography-mass spectrometry (GC-MS), we quantified various VLC fatty acids, primary alcohols, aldehydes, alkanes and 1-alkanol esters common to most plant species [30]. Furthermore, benzyl esters, phenethyl esters, *p*-hydroxyphenethyl esters were identified for the first time in wheat wax, together with various terpenoids. Finally, the polyketides characteristic of Poaceae waxes were reported, including β-diketones, hydroxy-β-diketones, alkylresorcinols, methyl alkylresorcinols and 2-alkanol esters. However, numerous compounds in the cuticular wax mixtures of wheat flag leaf blades and peduncles remained unidentified. To elucidate the molecular structures of these additional wheat wax constituents, we have now performed in-depth mass spectrometric analyses, using various derivatives of each structure for comparison of fragmentation patterns. Finally, the homolog and isomer patterns of the identified compound classes were assessed, to enable inferences on their biosynthetic origins.

## Methods

### Plant material

Flag leaves and peduncles were harvested from mature *Triticum aestivum* cv. Bethlehem plants during the months of August 2013 for total wax specimens and August 2014 for specimens for preparative thin layer chromatography. Plants were grown in greenhouses at Weizmann Institute of Science (Rehovot, Israel) on 50% peat– 50% turf, with watering every 3–4 days (~400 mL per 5 L pot). Growth conditions were: 12–14 h / 10–12 h light / dark cycles (180 μmol m^-2^ s^-1^ light), with temperatures of 24–26°C / 17–18°C, respectively. For total wax samples, one leaf blade with total area of 40–50 cm^2^ (both sides) and one peduncle with a projected surface area of ~20 cm^2^ were used per biological replicate. For preparative TLC samples, ten leaf blades and ten peduncles of the same size were used. Exact areas for leaf blades were determined by capturing them in photographs and using the ImageJ software to measure the area of one side, then multiplying by a factor of 2. For peduncles, areas were determined by measuring the length L and diameter D of the peduncle and then calculating the area of specimen with the formula: π x D x L.

### Chemicals

The following chemicals were acquired from Sigma-Aldrich (Oakville ON, Canada) and used without further purification: chloroform (≥99%, with 0.75% ethanol as stabilizer), ethanol (≥99.8%, HPLC grade), pyridine (≥99.8%, anhydrous), *N*,*O*-bis(trimethylsilyl)trifluoroacetamide (BSTFA, GC grade), acetic anhydride (≥98%), diethyl ether (≥99.7%, anhydrous, 1 ppm BHT as inhibitor), lithium aluminum hydride (≥95%), sulphuric acid (95–98%), *O*-methylhydroxylamine hydrochloride (≥98%), boron trifluoride-methanol solution (14%), primuline (50% dye content), acetone (≥99.9%, HPLC grade). *n*-Tetracosane (≥99%) was from Alfa Aesar (Ward Hill MA, USA). Gases were acquired from Praxair Canada (Vancouver BC, Canada): nitrogen (≥99.998%), helium (≥99%), and hydrogen (≥99.95%).

### Preparation of wax extracts

Rolled-up leaves and peduncle pieces were extracted for 30 s at ambient temperature with 10 mL chloroform, to which 5 μg *n*-tetracosane were added prior to extraction, as an internal standard. The chloroform was then transferred to another vial and the extraction repeated for another 30 s with a fresh portion of 10 mL chloroform. The combined chloroform extracts were then evaporated to dryness under a stream of N_2_ at 50°C, leaving behind the wax mixtures for either preparative TLC or GC analysis.

### Preparative thin layer chromatography

Fractionation of compound classes in the total wax extracts was carried out by preparative TLC, using the sandwich technique [40]. Glass plates coated with silica gel 60 F_254_ (Uniplate_,_ Analtech, layer thickness: 1 mm, size: 20x20 cm, with 4 cm concentrating zone) served as stationary phase, and a mixture of chloroform:ethanol 98:2 (v/v) served as mobile phase. At the end of separation, TLC plates were sprayed with primuline (5 mg in 100 mL acetone/water 80/20, v/v) and bands were visualized under 365 nm UV light. All bands were removed from the plate with clean spatulas into several glass vials, and each extracted twice with 10 mL portions of fresh chloroform for 30 s, at ambient temperature. Then, the combined extracts were filtered through glass wool (Supelco), partially evaporated under N_2_ at 50°C, transferred to 2 mL GC vials, evaporated to dryness and stored until GC-Mass Spectrometry (MS) analysis.

### Derivatization reactions

Prior to GC analysis, all samples were silylated by refluxing with 10 μL *N*,*O*-bis(trimethylsilyl)trifluoroacetamide (BSTFA) and 10 μL pyridine at 70°C for 20 min. Excess reagents were then removed under a gentle stream of N_2_ and the silylated waxes re-dissolved in 50 μL CHCl_3_.

Acetylation was carried out by refluxing a mixture of dry wax, 10 μL pyridine, and 10 μL acetic anhydride at 70°C for 5 min, then allowing it to stir overnight at ambient temperature. After removal of excess reagents under N_2_, silylation was carried out as described above.

Complete reduction of carbonyl and ester groups was achieved by dissolving the wax sample in 50 μL diethyl ether and adding 0.1 mg LiAlH_4_, then allowing the mixture to react overnight at 70°C. After quenching with 10% H_2_SO_4_, followed by three sequential extractions with 60 μL diethyl ether each, the combined extracts were evaporated to dryness and silylated as described above.

Carbonyl-containing compounds were transformed into their methoximes by heating with 20 μL of a saturated solution of *O*-methylhydroxylamine hydrochloride in pyridine:chloroform 7:3 (v/v) for 30 min at 70°C. The resulting mixture was partitioned between 50 μL distilled water and 50 μL chloroform and the chloroform fraction retained. After extracting the aqueous phase one more time with 50 μL fresh chloroform, the chloroform extracts were combined, evaporated to dryness and silylated as described above.

Transesterification was achieved by heating the waxes in 100 μL of 14% BF_3_-methanol solution at 70°C for 2 hours. Then, the products were isolated by partitioning between 50 μL distilled water and 50 μL diethyl ether and repeating the extraction two more times with fresh portions of 50 μL ether. After evaporation to dryness, silylation was carried out as described above.

### Gas chromatography

Two Gas Chromatography (GC) instruments were used for identification and quantification of wax constituents, respectively, both equipped with the same type of capillary GC column (6890N, Agilent, Avondale PA, USA; length: 30 m; type: HP-1 100% PDMS; i.d.: 0.32 mm; df: 0.1 µm), both equipped with on-column injector and programmed to follow the same temperature program (2 min at 50°C, ramp 40°C min^-1^ to 200°C, constant for 2 min, ramp 3°C min^-1^ to 320°C, constant for 30 min). One GC instrument employed helium as mobile phase, at a flow rate of 1.4 mL/min, and was equipped with MS detector (5973N, Agilent, EI-70 eV, *m/z* 50–750). The other used hydrogen as carrier gas at 2.0 mL/min and was equipped with a flame ionization detector (FID). Wax compounds were quantified by normalizing their GC-FID peak areas against that of the internal standard, added in known amount. The relative response factors of all wax compound classes with respect to the internal standard were approximated to 1.00, in agreement with literature reports using the same GC-FID operation conditions [41]. Relative abundances of hydroxyl α-fragments in selected GC-MS runs were used to quantify isomer distributions within single homologs of compounds bearing (TMS-derivatized) secondary OH-groups as described by Wen and Jetter (2009). Similarly, relative abundances of secondary cations generated by cleavage of C-C bonds next to methyl branches were used to quantify isomer distributions within homologs of branched alkanes.

## Results

The principal goals of the work herein were to identify compounds with secondary functional groups in the cuticular waxes of the bread wheat (*Triticum aestivum*) cultivar Bethlehem (sections 3.1.– 3.4.) and to determine their relative quantities within the wax mixtures covering flag leaf blades and peduncles (section 3.5.).

### TLC separation of cuticular waxes of T. aestivum flag leaf blades and peduncles

Preliminary experiments showed that the unknown wheat wax constituents belonged to seven different compound classes **A**-**G**. Each of these comprised a series of compounds separated into equally spaced GC peaks with shared characteristic MS fragmentation patterns, and thus each class was recognized as a homologous series of compounds. To enable their structure elucidation, the compound classes were separated and concentrated by preparative thin layer chromatography (TLC) using silica gel as stationary phase and CHCl_3_:EtOH 98:2 (v/v) as mobile phase.

The flag leaf blade wax mixture was separated into eleven fractions, which were analyzed individually by GC-MS. Among them, seven fractions were found to contain previously identified compound classes, namely 5-alkylresorcinols and methyl 5-alkylresorcinols (R_f_ 0.09), 2-(*p*-hydroxyphenyl)ethyl esters of VLC fatty acids (R_f_ 0.27), free VLC fatty acids (R_f_ 0.30), hydroxy-β-diketones (R_f_ 0.33), 1-alkanols (R_f_ 0.38), β-diketones along with small amounts of aldehydes (R_f_ 0.86), and several very non-polar wax classes such as *n*-alkanes, *iso-* and *anteiso-*alkanes, esters of 1- and 2-alkanols, benzyl esters and 2-phenylethyl esters (R_f_ 1.00). The unknown compound classes were found to have widely varying TLC behaviour, with series **A** (R_f_ 0.72), **C** (R_f_ 0.54), **D** (R_f_ 0.35), as well as **E** and **F** (R_f_ 0.44) in fractions of their own, and series **B** co-eluting with free VLC fatty acids (R_f_ 0.30) and series **G** with the non-polar wax classes (R_f_ 1.00). The peduncle wax mixture yielded nine fractions, with identical R_f_ values and compositions very similar to corresponding leaf wax fractions. However, series **A**, **B** and **C** were not found in peduncle wax.

### Structure elucidation of compound classes A–C

**Fig 1:**
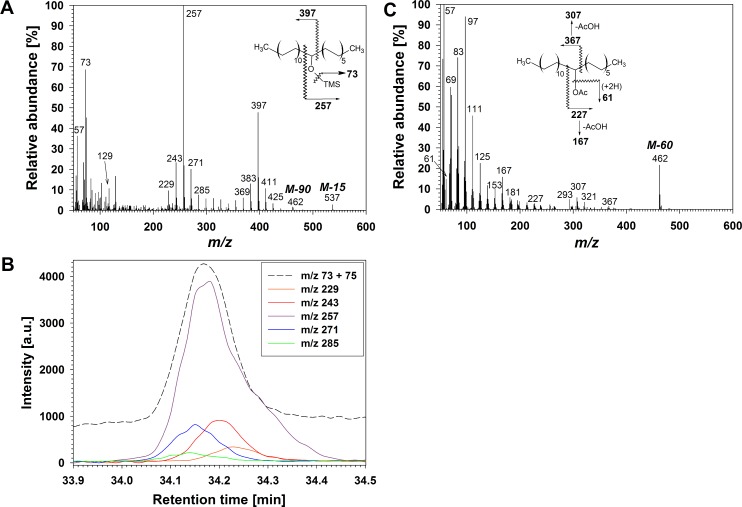
Structure elucidation of secondary alcohols in wheat leaf wax. (A) Mass spectrum of co-eluting TMS derivatives of C_33_ *sec* alcohol isomers and major fragmentations of main isomer. (B) EICs showing cumulative intensity of *m/z* 73 and 75, as well as intensities of short α-fragments of main isomer and the four next most abundant isomers. a.u.: arbitrary units. (C) Mass spectrum of co-eluting Ac derivatives of C_33_ *sec* alcohol isomers and major fragmentations of main isomer.

Based on TLC behaviour, fraction **A** exhibited polarity intermediate between aldehydes and primary alcohols, and it was thus hypothesized to contain VLC secondary alcohols. All six compounds in **A** yielded TMS derivatives with common diagnostic MS fragment *m/z* 73 [(CH_3_)_3_Si]^+^, homolog-dependent M-15 and M-90 ions, due to loss of methyl radical and (CH_3_)_3_SiOH, respectively, and no *m/z* 147 [(CH_3_)_2_SiOSi(CH_3_)_3_]^+^ (Fig 1A), together confirming the secondary alcohol structure [15,19,42]. Each homolog, in its TMS derivative mass spectrum, also displayed a pair of α-fragments, with one ion *m/z* 257 common to all homologs indicative of a hydroxyl group on C-12, and a second fragment varying with chain length (*m/z* 397 for the C_33_ homolog in Fig 1A). These fragments were accompanied by further pairwise combinations of α-fragments differing by 14 amu units (*e*.*g*., C-10 *m/z* 229, C-11 *m/z* 243, C-13 *m/z* 271, C-14 *m/z* 285). A summary of all identified homologs and regiomers within them together with their diagnostic MS fragments is presented in S1 Table. Extracted ion chromatograms (EICs) of the shorter α-fragments in these pairs revealed small retention time differences between them, but all well within the overall GC peak of the respective homolog. Taken together, the GC and MS information thus revealed the presence of positional isomers (regiomers) of secondary alcohols that were only partially GC-separated. All six homologs were found dominated by the C-12 isomer, accompanied by others bearing hydroxyls on neighbouring carbon atoms (Fig 1B).

For structure confirmation, acetyl (Ac) derivatives of compounds **A** were prepared and analyzed by GC-MS, revealing a characteristic fragment *m/z* 61 [CH_3_COOH_2_]^+^ common to all homologs indicative of a hydroxyl group [12,15], as well as M-60 fragments varying between homologs due to elimination of acetic acid AcOH (Fig 1C). α-fragments were much less prominent than for TMS derivatives, but further loss of AcOH resulted in distinct pairs of fragments confirming the presence of different regiomers, with *m/z* 167 / 307 for the C-12 alcohol isomer, *m/z* 153 / 321 for the C-11 isomer, *m/z* 181 / 293 for C-13, etc. Taken together, the TLC behaviour and GC-MS data for TMS and Ac derivatives demonstrated that **A** was a series of secondary alcohol homologs, each comprising several regiomers with hydroxyls predominantly at C-12 but also on adjacent carbons (S1 Table).

**Fig 2:**
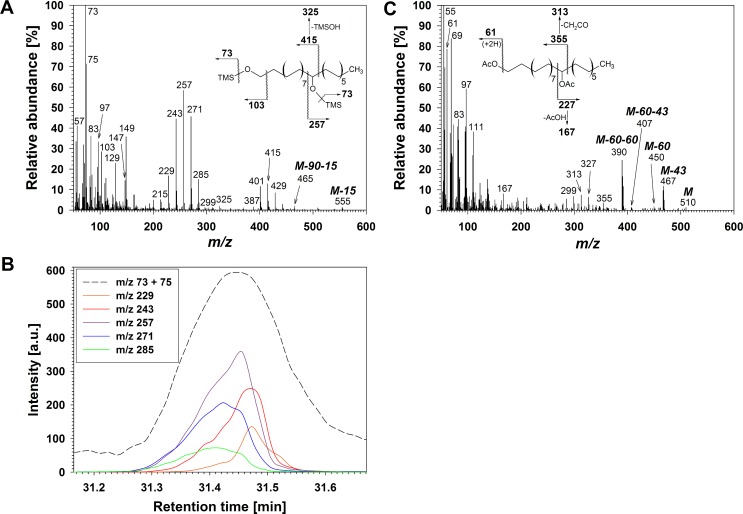
Structure elucidation of primary/secondary diols in wheat leaf wax. (A) Mass spectrum of co-eluting TMS derivatives of C_28_ *prim/sec* diol isomers and major fragmentations of main isomer. (B) EICs showing intensities of m/z 73 and of short α-fragments of main isomer and the four next most abundant isomers. a.u.: arbitrary units. (C) Mass spectrum of co-eluting Ac derivatives of C_28_ *prim/sec* diol isomers and major fragmentations of main isomer.

Series **B** consisted of three compounds found in the same fraction as free fatty acids in flag leaf wax. The TMS derivatives of all compounds in **B** shared diagnostic MS fragments *m/z* 73 [(CH_3_)_3_Si]^+^ and *m/z* 75 [(CH_3_)_2_SiOH]^+^, *m/z* 103 [(CH_3_)_3_SiOCH_2_]^+^, and *m/z* 147 [(CH_3_)_2_SiOSi(CH_3_)_3_]^+^ and *m/z* 149 [(CH_3_)_2_SiOSi(CH_3_)_2_OH]^+^, together indicating the presence of one primary and one secondary hydroxyl group (Fig 2A) [13,18,43]. They were accompanied by homolog-dependent M-15 and M-15-90 ions, due to loss of methyl radical and loss of both methyl and (CH_3_)_3_SiOH, respectively. Pairs of α-fragments, including one ion *m/z* 257 common to all homologs and a second fragment varying with chain length (*m/z* 415 for the C_28_ homolog in Fig 2A), indicated a hydroxyl group on the ω-12 carbon. Further α-fragments differing by 14 amu were present, and EICs revealed small retention time differences between them (Fig 2B). The diagnostic MS fragments for all identified homologs and regiomers are presented in S2 Table.

The Ac derivatives of compounds in fraction **B** showed a characteristic fragment *m/z* 61 [CH_3_COOH_2_]^+^ confirming the presence of at least one hydroxyl group (Fig 2C), as well as homolog-dependent parent ions M and daughter ions due to loss of up to two acetyl groups and acetic acid molecules (M-43, M-60, M-60-43 and M-60-60), supporting the presence of a second hydroxyl functionality [15]. Different regiomers could be discerned based on loss of CH_2_CO or AcOH from α-fragments, confirming the presence of a hydroxyl function on ω-12 (*m/z* 167 / 313) and, for example, ω-11 (*m/z* 153 / 327), ω-13 (*m/z* 181 / 299). Taken together, the TLC behaviour and GC-MS data for TMS and Ac derivatives demonstrated that **B** was a homologous series of *prim*/*sec* diols, with *sec* hydroxyl groups predominantly in the ω-12 position or on adjacent carbons (S2 Table).

**Fig 3:**
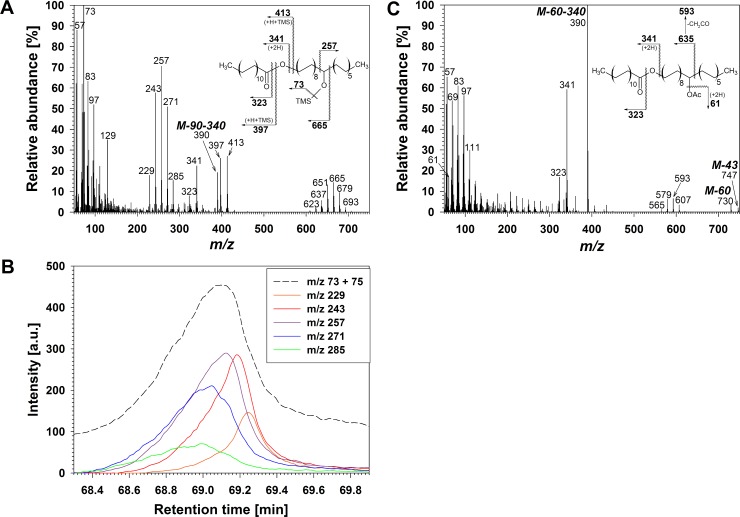
Structure elucidation of primary/secondary diol esters in wheat leaf wax. (A) Mass spectrum of co-eluting TMS derivatives of C_50_ *prim/sec* diol ester isomers. (B) EICs showing intensities of *m/z* 73 and of short α-fragments of main isomer and the four next most abundant isomers. a.u.: arbitrary units. (C) Mass spectrum of co-eluting Ac derivatives of C_50_ *prim/sec* diol ester isomers. (D) Major fragmentations of main isomer from (A) and from (C).

Series **C** comprised five compounds with very long GC retention times (>50 min), suggesting relatively high molecular weights and long carbon chains, likely in the form of esters linking two VLC moieties. Based on TLC behaviour, these compounds had polarities between primary and secondary alcohols, rendering alkyl ester structures with an additional secondary hydroxyl function plausible. The TMS derivatives of compounds **C** had a common diagnostic MS fragment *m/z* 73 [(CH_3_)_3_Si]^+^, but no *m/z* 147 [(CH_3_)_2_SiOSi(CH_3_)_3_]^+^ (Fig 3A), indicating the presence of only one hydroxyl group in the native compounds. The five homologs also had fragments characteristic of the acid components of esters, such as acylium ions M_acid_-17 and fragments formed via McLafferty rearrangement with double hydrogen transfer M_acid_+1 (*m/z* 323 and 341 for the C_50_ homolog containing C_22_ acid shown in Fig 3A) [44]. The hydroxy ester structure thus confirmed, further ions could be inferred to result from TMS transfer from the *sec* hydroxyl to the ester group, i.e. *m/z* 397 and 413 for the C_50_ homolog in Fig 3A [20,45], and from sequential loss of (CH_3_)_3_SiOH and an acid molecule from M (C_22_ acid with molecular weight 340 amu in Fig 3A).

The TMS derivatives of **C** also showed pairs of α-fragments, one common to all homologs (*m/z* 257) indicating an ω-12 hydroxyl group, and another one varying with chain length (*m/z* 665 for the C_50_ homolog in Fig 3A). Other pairs of α-fragments differing by 14 amu units again suggested positional isomers, and small retention time differences in EICs confirmed the presence of, among others, ω-10 (*m/z* 229), ω-11 (*m/z* 243), ω-13 (*m/z* 271), ω-14 (*m/z* 285) hydroxyls. (Fig 3B; S3 Table). Ac derivatives served to confirm the tentatively assigned structures of **C**, with fragments *m/z* 61 [CH_3_COOH_2_]^+^ as well as M-43 and M-60 fragments due to loss of an acetate moiety corroborating the presence of one hydroxyl function in all homologs (Fig 3C). Further fragments due to ester-linked acids (M_acid_-17 and M_acid_+1), to loss of both acetic acid and the VLC acid from M (*m/z* 390) further confirmed the hydroxy ester structures. Finally, α-fragmentation and subsequent loss of CH_2_CO gave rise to ions confirming the presence of an ω-12 hydroxyl group (*m/z* 593), together with several co-eluting positional isomers (*e*.*g*., *m/z* 607 for ω-11 and *m/z* 579 for ω-13 hydroxyls).

Reduction of a separate aliquot of series **C** with excess lithium aluminum hydride (LAH) yielded a single bifunctional compound, C_28_ *prim/sec* diol, together with C_16_ to C_24_ *prim* alcohols. The structure of the C_28_ *prim/sec* diol was assigned based on identical GC and MS behaviour (of the TMS derivative) to the corresponding homolog in series **B**, thus confirming that compounds **C** were esters of *prim*/*sec* C_28_ diols. Taken together, our TLC and GC-MS data demonstrate that fraction **C** was a homologous series of esters containing C_16_ to C_24_ fatty acids linked to the terminal hydroxyl of *prim*/*sec* C_28_ diols. Each ester homolog comprised several isomers, with *sec* hydroxyl groups at and around the ω-12 carbon. The C_28_ diol resulting from reduction of fraction C comprised ca. 30% of the ω-12 isomer, 25% each of ω-11/13, 10% each of ω-10/14, and 1% of further, adjacent isomers.

### Structure elucidation of compound classes D and E

**Fig 4:**
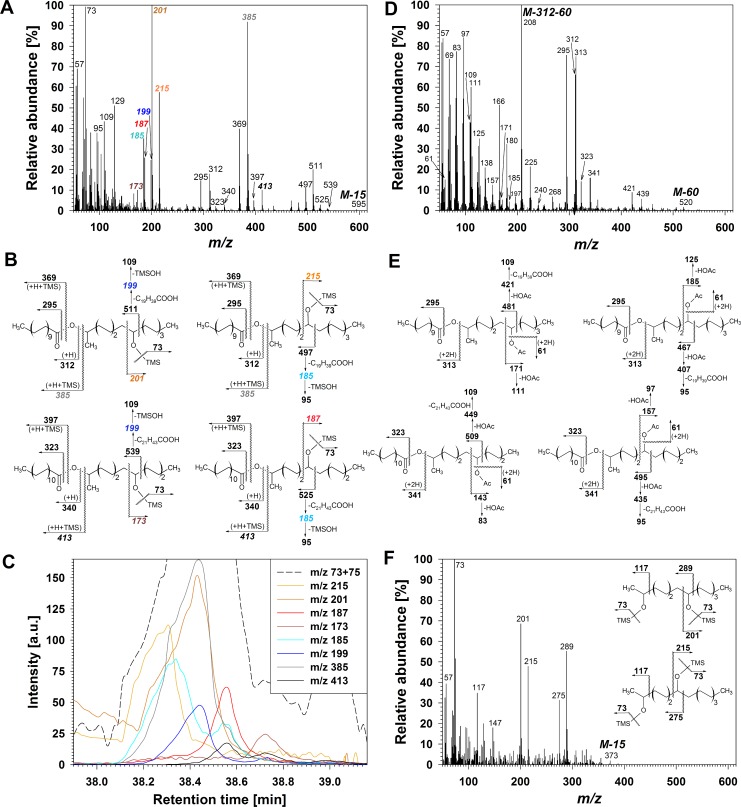
Structure elucidation of hydroxy-2-alkanol esters in wheat leaf and peduncle wax. (A) Mass spectrum of co-eluting TMS derivatives of C_35_ hydroxy-2-alkanol ester isomers. (B) Major fragmentations of all isomers in (A). (C) EICs showing cumulative intensity of *m/z* 73 and 75, as well as intensities of: metamer-shared α-fragments *m/z* 185 and 199, regiomer-characteristic α-fragments *m/z* 173, 187, 201 and 215, and TMS-transfer acid fragments *m/z* 385 and 413. a.u.: arbitrary units. (D) Mass spectrum of co-eluting Ac derivatives of C_35_ hydroxy-2-alkanol ester isomers. (E) Major fragmentations of all isomers in (D). (F) Mass spectrum and major fragmentations of TMS derivatives of co-eluting C_15_ diol isomers obtained via LiAlH_4_ reduction of the C_35_ hydroxy-2-alkanol ester isomer mixture (corresponding information for C_13_ diol isomers not shown).

The five homologs in series **D** were, according to relative TLC retention, more polar than primary alcohols and the hydroxy esters in fraction **C**. Compounds **D** were tentatively assigned as VLC esters of hydroxy-2-alkanols linked through the 2-OH group, based on similarity of their TMS derivative mass spectra with that of 7-hydroxypentadecan-2-ol eicosanoate reported in the literature [45]. The combination of TMS derivative M-15 and ion *m/z* 73 [(CH_3_)_3_Si]^+^ together with the lack of *m/z* 147 [(CH_3_)_2_SiOSi(CH_3_)_3_]^+^ confirmed the presence of only one hydroxyl function. Two ester metamers with differing acid (and, consequently, also alcohol) chain lengths were immediately apparent for each homolog in **D**, based on the presence of two homologous pairs of M_acid_-17 / M_acid_ fragments (*m/z* 295 / 312 and 323 / 340 for the C_35_ homolog in Fig 4A). Similarly, homologous pairs of TMS transfer fragments were observed (*m/z* 369 / 385 and 397 / 413 in Fig 4A), indicative of two co-eluting metamers for each ester homolog. Furthermore, four pairs of complementary α-fragments were observed for each ester chain length (*m/z* 215 / 497, 201 / 511, 187 / 525, and 173 / 539), suggesting the presence of two regiomers for each of the two metamers. Careful analysis of overlap between EIC traces of regiomer-specific α-fragments and of acid-specific fragments (Fig 4C) allowed identification of four co-eluting isomers within the C_35_ ester homolog, listed in decreasing relative abundance: (i) C_20_ acid + C_15_ 2,8-diol (*m/z* 385 and 201); (ii) C_20_ acid + C_15_ 2,7-diol (*m/z* 385 and 215); (iii) C_22_ acid + C_13_ 2,7-diol (*m/z* 413 and 187); (iv) C_22_ acid + C_13_ 2,8-diol (*m/z* 413 and 173). A summary of all identified homologs and isomers within them along with their diagnostic MS fragments is presented in S4 Table.

The structure of the diol esters in **D** were confirmed by MS analysis of their Ac derivatives. They showed the characteristic *m/z* 61 [CH_3_COOH_2_]^+^ indicative of hydroxyl group presence in the native structure, as well as homolog-dependent M-60, M-M_acid_, M-M_acid_-43, M-M_acid_-60 fragments due to single or combined losses of acetyl- and fatty acyl-derived moieties (Fig 4D). Fragments M_acid_-17 / M_acid_+1 further confirmed the presence of two metamers per homolog (*m/z* 295 / 313 and 323 / 341 in Fig 4D). The α-fragments had very low intensity, but their daughter ions resulting from loss of acetic acid confirmed the presence of the *sec* hydroxyl (*m/z* 111 and 421 in Fig 4D). Finally, reduction of an aliquot of the fraction with excess LAH gave rise to two new compounds with relatively short GC retention times (as TMS derivatives). Their mass spectra could be unambiguously assigned to mixtures of pentadecane-2,7-diol plus pentadecane-2,8-diol (Fig 4F) and tridecane-2,7-diol plus tridecane-2,8-diol, respectively, thus confirming the presence of four isomers per ester homolog in **D**. Taken together, the TLC and GC-MS data identified **D** as a series of ester homologs formed by linking various fatty acids with 7- and 8-hydroxy-2-tridecanol as well as 7- and 8-hydroxy-2-pentadecanol. The 7- and 8-hydroxy-2-alkanol isomers were found in approximately equal amounts overall.

**Fig 5:**
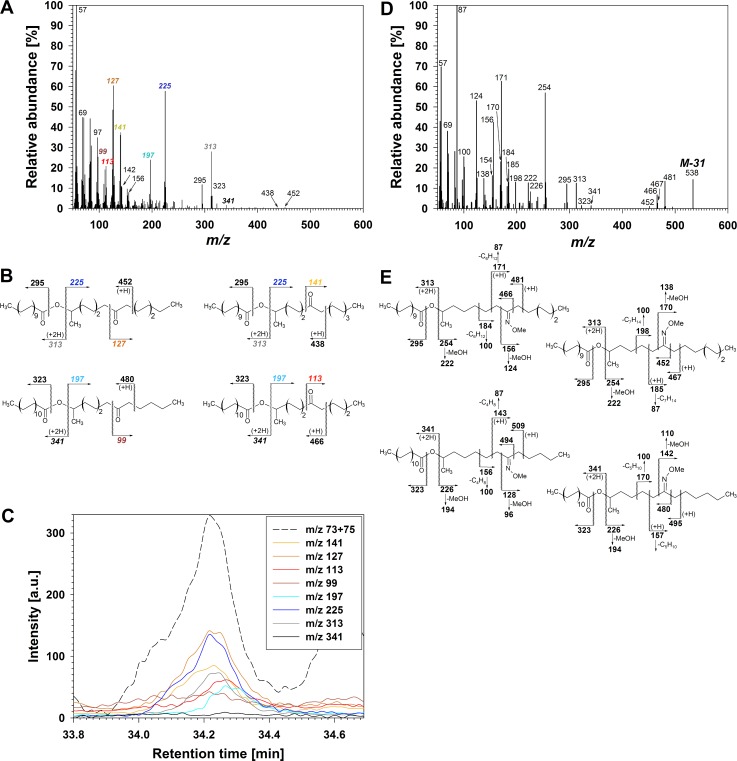
Structure elucidation of oxo-2-alkanol esters in wheat leaf and peduncle wax. (A) Mass spectrum of co-eluting isomers of C_35_ oxo-2-alkanol ester. (B) Major fragmentations of all isomers from (A). (C) EICs showing intensity of *m/z* 57, as well as intensities of: metamer-characteristic alkyl fragments *m/z* 197 and 225, regiomer-characteristic α-fragments *m/z* 99, 113, 127 and 141, and M_acid_+1 fragments 3 *m/z* 13 and 341. a.u.: arbitrary units. (D) Mass spectrum of co-eluting isomers of methoxime derivatives of C_35_ oxo-2-alkanol ester. (E) Major fragmentations of all isomers from (D).

The five homologs in series **E**, with polarity between primary alcohols and *prim/sec* diol esters, were tentatively assigned oxo-2-alkanol ester structures based on similarity of their mass spectra with that of 7-oxopentadecan-2-ol eicosanoate reported before [45]. Treatment of **E** with BSTFA left the compounds in **E** lacking the TMS fragment *m/z* 73, suggesting that they did not bear hydroxyl groups (Fig 5A). Instead, they had fragments indicative of the 2-alkanol ester structure, such as M_acid_-17 (*m/z* 295 and 323 for the two metamers in Fig 5A), M_acid_+1 (*m/z* 313 and 341 in Fig 5A), and M_alcohol_-1 (*m/z* 225 and 197 in Fig 5A). They also showed prominent homolog-independent α-fragments indicating the carbonyl position on the 2-alkanol moiety, either 7- or 8-oxo groups on C_15_ 2-alkanol (*m/z* 141 and *m/z* 127, respectively), and 7- or 8-oxo groups on C_13_ 2-alkanol (*m/z* 113 and *m/z* 99, respectively; Fig 5A–5C and S5 Table). Complementary α-fragments were not observed, but some of the closely-related fragments resulting from McLafferty rearrangement on the same side of the carbonyl function were sizeable (*m/z* 452 and 438 for the C_35_ homolog in Fig 5A). Molecular ions M could not be detected under the current conditions.

To directly probe the presence of a carbonyl functionality, an aliquot of fraction **E** was derivatized with *O-*methylhydroxylamine and the corresponding methoximes analyzed by MS. The resulting homologs all showed fragments *m/z* 87 and *m/z* 100 diagnostic for methoximes [18] (Fig 5D), accompanied by prominent MS ions characterizing the 2-alkanol ester structure, such as M_acid_-17 (*m/z* 295 and 323 for the two metamers in Fig 5D), M_acid_+1 (*m/z* 313 and 341 in Fig 5D), and M_alcohol_-1 (*m/z* 254 and 226 in Fig 5D). α-Fragments and related McLafferty fragments indicative of methoxime position were 29 amu higher than corresponding signals of the underivatized carbonyls (*e*.*g*., *m/z* 156 and *m/z* 481 in Fig 5D instead of *m/z* 127 and *m/z* 452 in Fig 5A). Finally, fragments M-31 due to loss of methoxy group indicated homolog chain lengths, while M ions were not detected.

For further structure confirmation fraction **E** was subjected to reduction with LAH, resulting in the same two pairs of diol isomers also formed by reduction of series **D**. Taken together, the TLC behaviour as well as the GC-MS characteristics of diverse derivatives demonstrated that fraction **E** was a homologous series of esters containing various fatty acids linked to 7- and 8-oxo-2-tridecanol (IUPAC names: 12-hydroxytridecan-7-one and 12-hydroxytridecan-6-one) as well as 7- and 8-oxo-2-pentadecanol (IUPAC names: 14-hydroxypentadecan-9-one and 14-hydroxypentadecan-8-one)_._ Across all homologs, the 7- and 8-oxo-2-alkanol isomers were found in approximately equal amounts overall.

### Structure elucidation of compound classes F and G

**Fig 6:**
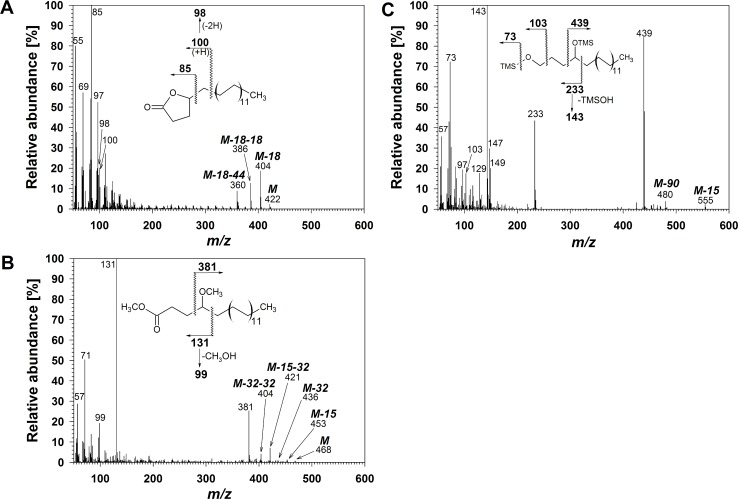
Structure elucidation of 4-alkylbutan-4-olides in wheat leaf and peduncle wax. (A) Mass spectrum and major fragmentations of C_28_ 4-alkylbutan-4-olide. (B) Mass spectrum and major fragmentations of its product of transesterification with CH_3_OH/BF_3_. (C) Mass spectrum and major fragmentations of TMS derivative of LiAlH_4_ reduction product from C_28_ 4-alkylbutan-4-olide.

Series **F** comprised four compounds, found in the same fraction as series **E**, tentatively identified as VLC 4-alkylbutan-4-olides (4-alkyl-γ-lactones) based on similarity of their mass spectra with those of shorter-chain 4-alkylbutan-4-olides reported previously [46]. Similar to series **E**, compounds **F** could not be silylated (no *m/z* 73), suggesting that they lacked a hydroxyl group (Fig 6A). All homologs had a diagnostic base peak *m/z* 85, likely formed via cleavage of the alkyl side chain, as well as *m/z* 100 formed via McLafferty rearrangement. Chain length-dependent molecular ions were accompanied by fragments M-18, M-18-18 and M-18-44, likely due to loss of water and acetaldehyde (S6 Table).

Transesterification of **F** with excess CH_3_OH/BF_3_ resulted in an open-chain product that could not be silylated, similar to the behaviour of 5-alkyl-δ-lactones under the same conditions [47], albeit independent of derivatization time. The transesterification products of **F** were identified as methyl 4-methoxyalkanoates based on their shared α-fragment *m/z* 131, and a second homolog-dependent α-fragment (*m/z* 381 for the C_28_ homolog in Fig 6B). Molecular ions were found accompanied by M-15, M-32, M-15-32, and M-32-32 due to loss of methyl radical and/or methanol molecule(s). Lastly, reduction of **F** with excess LAH followed by reaction with BSTFA resulted in silylated 1,4-diols with MS characteristics (Fig 6C) indicative of two hydroxyl groups (*m/z* 73 [(CH_3_)_3_Si]^+^, *m/z* 75 [(CH_3_)_2_SiOH]^+^, *m/z* 103 [(CH_3_)_3_SiOCH_2_]^+^, *m/z* 147 [(CH_3_)_2_SiOSi(CH_3_)_3_]^+^ and *m/z* 149 [(CH_3_)_2_SiOSi(CH_3_)_2_OH]^+^). Other diagnostic signals were the α-fragments *m/z* 233 (independent of homolog) and *m/z* 439 (depending on the homolog), along with the base peak *m/z* 143 resulting from loss of (CH_3_)_3_SiOH from the shorter α-fragment. Chain length-dependent M-15 (loss of methyl) and M-90 (loss of (CH_3_)_3_SiOH) further confirmed the 1,4-diol structures of the reduction products. All data for **F** taken together unambiguously established this as a homologous series of 4-alkylbutan-4-olides (4-alkyl-γ-lactones).

**Fig 7:**
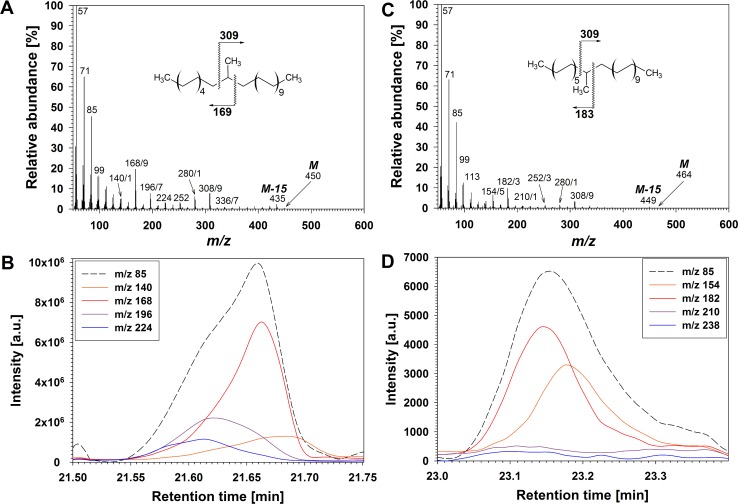
Structure elucidation of internally branched alkanes in wheat leaf and peduncle wax. (A) Mass spectrum of co-eluting isomers of C_32_ internally branched alkane and major fragmentations of the main isomer. (B) EICs showing intensities of the alkyl fragment *m/z* 85 and of regiomer-characteristic α-fragments *m/z* 140, 168, 196 and 224. a.u.: arbitrary units. (C) Mass spectrum of co-eluting isomers of C_33_ internally branched alkane and major fragmentations of the main isomer. (B) EICs showing intensities of the alkyl fragment *m/z* 85 and of regiomer-characteristic α-fragments *m/z* 154, 182, 210 and 238.

Series **G** comprised six compounds in the least polar fraction of wheat wax, tentatively assigned as internally methyl-branched alkanes by analogy with spectra of 15-methyl-alkanes [48]. Accordingly, compounds **G** did not exhibit MS fragments indicative of silylation, and other derivatization reactions (such as acetylation, methoximation, transesterification or LAH reduction) did not alter the compounds in any way, thus confirming the absence of functional groups. Homolog-dependent molecular ions M and fragments M-15 (due to loss of the methyl branch) were observed (Fig 7A and 7C). They were accompanied by α-fragments diagnostic for the methyl branch position, with predominant *m/z* 168/169 and 196/197 common to all homologs with odd chain lengths (even total carbon numbers) and indicative of methyl branching at C-11 or C-13 (Fig 7A), and *m/z* 182/183 common to all even-numbered homologs (with odd total carbon numbers) and indicative of methyl branching at C-12 (Fig 7C). Complementary homolog-dependent α-fragments were *m/z* 308/309 and 280/281 (Fig 7A and 7C). Also noticeable were other α-fragments of additional, less abundant regiomers bearing methyl branches between C-9 (*m/z* 140/141) and C-16 (*m/z* 224/225) (Fig 7B and 7D). A summary of all in-chain branched alkane homologs and isomers identified here is presented in S7 Table together with their diagnostic MS fragments. Overall, our MS data identified compounds **G** as a homologous series of alkanes bearing methyl branches primarily on C-11/13 or C-12 depending on the parity of the homolog (S7 Table).

### Quantification of new compounds from cuticular waxes of *T*. *aestivum* cv. Bethlehem flag leaf blades and peduncles

**Fig 8:**
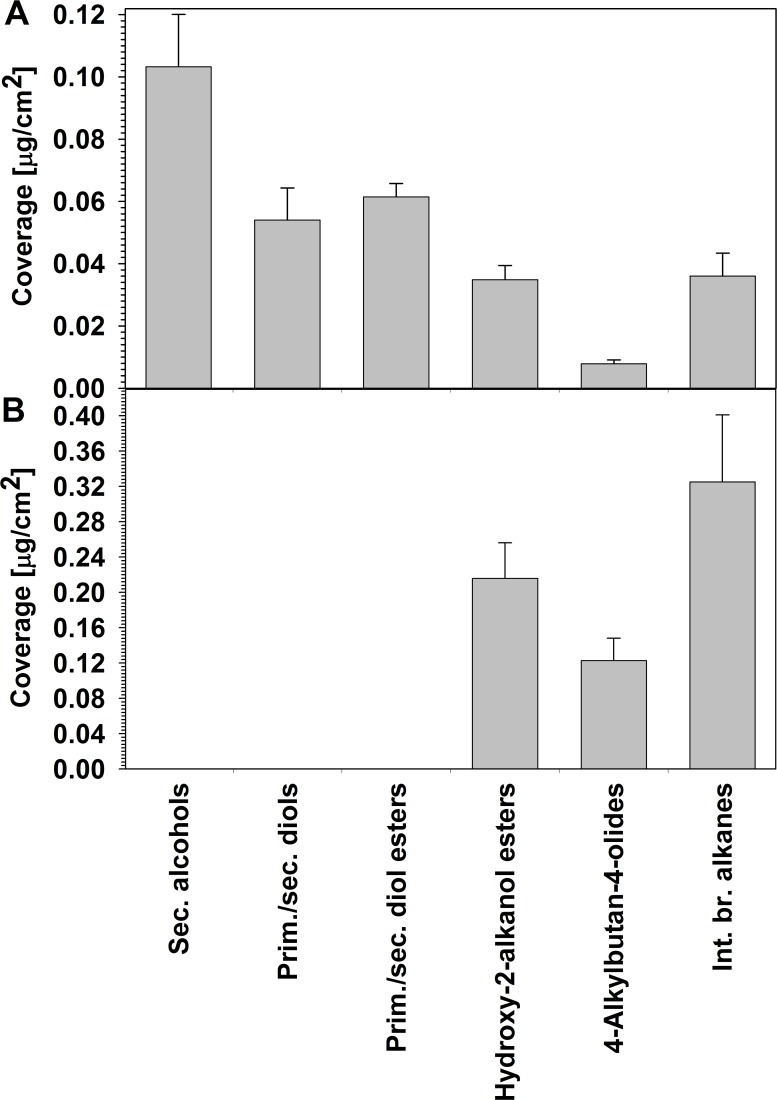
Total coverages of new compound classes in wheat leaf and peduncle waxes. Coverages (μg/cm^2^) of compound classes identified in the total wax mixtures covering the (A) flag leaf blade and (B) peduncle of *T*. *aestivum* cv. Bethlehem. Bars represent mean ± standard deviation (*n* = 5).

Gas chromatography with flame ionization detection (GC-FID) was used in a second set of experiments to quantify the newly identified compounds in the total wax mixtures of wheat flag leaves and peduncles. They had fairly low wax coverages over both organs, ranging from 0.008 ± 0.001 μg/cm^2^ for 4-alkylbutan-4-olides to 0.10 ± 0.02 μg/cm^2^ for secondary alcohols in the flag leaf waxes, and from 0.12 ± 0.03 μg/cm^2^ for 4-alkylbutan-4-olides to 0.32 ± 0.08 μg/cm^2^ for internally branched alkanes in peduncle waxes (Fig 8A and 8B). Neither of the oxo-2-alkanol ester homologs could be quantified reliably enough to calculate respective coverages.

**Fig 9:**
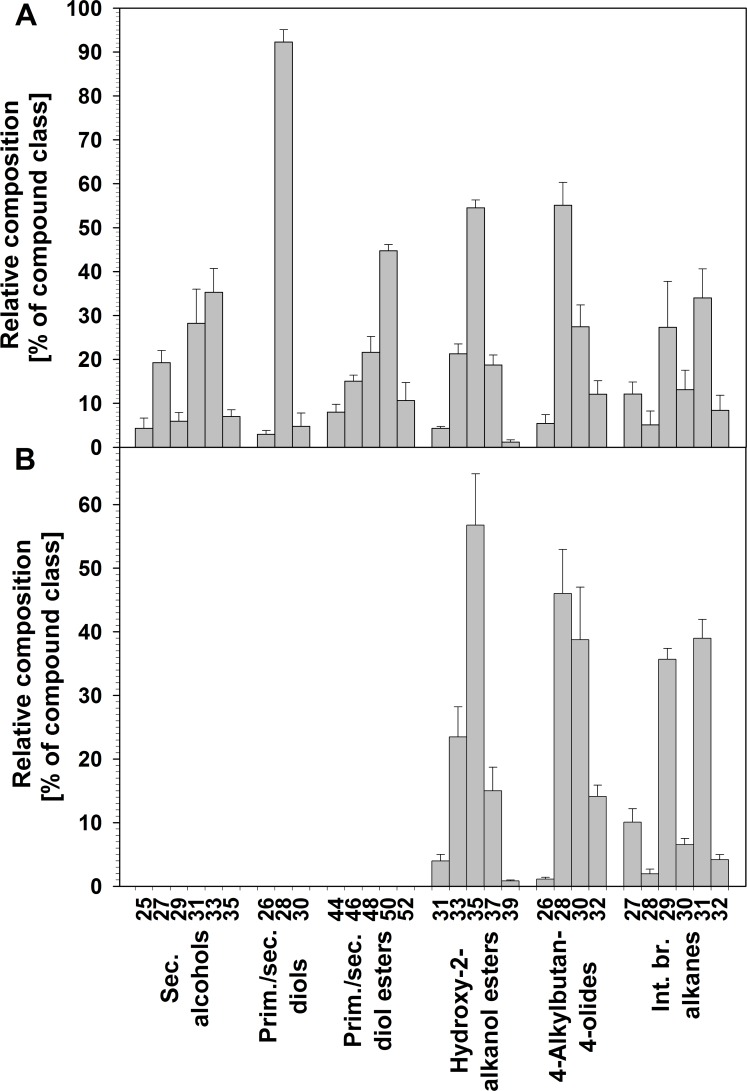
Chain length distributions of new compound classes in wheat leaf and peduncle waxes. Relative adundances (%) of each homolog from each of the six compound classes identified in the total wax mixtures covering the (A) flag leaf blade and (B) peduncle of *T*. *aestivum* cv. Bethlehem. Numbers on the *x*-axis indicate homolog chain length. Bars represent mean ± standard deviation (*n* = 5). Each bar group adds up to 100%.

As all new wheat wax compound classes comprised series of homologs, their characteristic chain length distributions could be further assessed. Secondary alcohols (compounds **A**) were found as a homologous series with odd-numbered carbon chains from C_25_ to C_35_ and a bimodal distribution peaking at C_27_ and C_33_ (Fig 9A). In contrast, the *prim/sec* diols (compounds **B**) ranged from C_26_ to C_30_, with only even-numbered homologs present and a single homolog, C_28_, accounting for more than 90% of this fraction. Similarly, the *prim/sec* diol esters (compounds **C**) contained the same alkyl moiety together with various fatty acids, resulting in a homologous series of even-numbered total chain lengths from C_44_ to C_52_ that peaked at C_50_. All three compound classes were found only in flag leaf wax.

Hydroxy-2-alkanol esters (compounds **D**) were present in both flag leaf and peduncle wax mixtures, with chain length profiles, spanning the odd-numbered homologs from C_31_ to C_39_ and a single maximum at C_35_ (Fig 9A and 9B). The fraction of oxo-2-alkanol esters (compounds **E**) comprised odd-numbered C_31_ to C_39_ homologs, peaking around C_35_.

The wheat waxes contained even-numbered C_26_ to C_32_ 4-alkylbutan-4-olides (compounds **F**) with unimodal chain length distributions peaking at C_28_ in both organs (Fig 9A and 9B). Finally, the internally branched alkanes (compounds **G**) had chain length profiles ranging from C_27_ to C_32_ in the wax mixtures from both organs, with odd-numbered chain lengths (even total carbon numbers) considerably more abundant than even-numbered chain lengths (with odd total carbon numbers). Among the odd-numbered chain lengths, the C_31_ homolog was most abundant, while C_30_ predominated among the even-numbered homologs.

## Discussion

Our in-depth analysis of the wax mixtures on flag leaf blades and peduncles of *T*. *aestivum* cv. Bethlehem revealed the presence of (i) homologous series of secondary alcohols, primary/secondary diols and primary/secondary diol esters, all with secondary hydroxyls on and around identical methylene units 12 carbons away from one chain end; (ii) esterified C_13_ and C_15_ 2-alkanols with hydroxyl- or keto-functions on C-7 or C-8; (iii) a homologous series of γ-lactones, as such derived from fatty acids with hydroxyl functions on C-4; and (iv) alkanes with (total) carbon numbers ranging from C_28_ to C_33_ and methyl branches on C-11 or C-12. Both the homolog and the isomer distribution of all seven compound classes can now be used to infer potential biosynthetic pathways leading to them.

### Secondary alcohols, primary/secondary diols and diol esters in flag leaf wax

The wax mixture on wheat flag leaves comprised homologous series of secondary alcohols, primary/secondary diols and corresponding diol esters, thus compounds characterized by secondary hydroxyls. All three compound classes were found to contain mainly regiomers with secondary hydroxyls on a methylene unit 12 carbons away from one end of the hydrocarbon chain, designated as C-12 in the secondary alcohols or as the ω-12 carbon in the diols. These major isomers in each of the three series were accompanied by further, minor regiomers, characterized by hydroxyls on methylene units in the vicinity of C-12. The finding that all three compound classes shared very similar isomer patterns around their secondary hydroxyl functions suggests that they are biosynthetically related.

The isomer distribution in all three compound classes, centred around one carbon position with minor but significant admixtures of isomers with hydroxyls on adjacent carbons is reminiscent of the isomer mixtures of wax secondary alcohols in several other species. For example, C_25_-C_33_ secondary alcohols with hydroxyls on C-6 to C-14 have been reported for leaf and fruit capsule waxes of several Papaveraceae species [49], C_29_-C_33_ secondary alcohols with hydroxyls on C-12 to C-17 for *Pisum sativum* leaf wax [15], and secondary alcohols, ketones, vicinal secondary/secondary diols and ketols with functional groups between C-13 and C-15 for *Arabidopsis thaliana* stem wax [14]. The latter Arabidopsis compounds are known to be formed by a single enzyme, the mid-chain alkane hydroxylase MAH1 [50]. This P450-dependent monooxygenase exhibits characteristically limited regio-specificity, catalyzing the (repeat) hydroxylation of several methylene units near the centre of the hydrocarbon chain. Accordingly, it has been proposed that homologous P450 enzymes with similarly limited regio-specificity form the broad mixtures of secondary alcohol isomers found in the waxes of other plant species [1].

It is important to note that a second important mechanism for introducing hydroxyl functions in wax molecules exists. In many plant species, wax secondary alcohols were identified with hydroxyl groups exclusively on even-numbered carbon atoms, such as 10-nonacosanol on *Malus domestica* fruit [51], 10-heptacosanol, 10-nonacosanol and 12-nonacosanol on *Osmunda regalis* fronds [52], or C_23_-C_33_ 2-alkanols from *Aloe arborescens* leaves [19]. It has been proposed that such secondary alcohols, with functional groups on every other rather than adjacent carbons, may be derived from β-hydroxyacyl-CoA intermediates of fatty acid elongation instead of P450 hydroxylation [17,19].

Our current findings of broad isomer distributions for the wheat secondary alcohols suggest that they may be synthesized by a P450 enzyme and not as derivatives of fatty acid elongation intermediates. We conclude that wheat likely possesses a MAH1-like enzyme hydroxylating preferentially on C-12 of C_25_-C_35_ alkane substrates. Interestingly, the resulting secondary alcohols had a bimodal chain length distribution, peaking at C_27_ and C_33_ (compare Fig 9A), very different from that of the corresponding alkane precursors, with a single maximum at C_31_ [30]. This suggests either an unusual chain length preference of this hydroxylase for C_33_ and C_27_ alkane substrates, or else the presence of two very similar enzymes with similar regio-specificity but different substrate chain length preference.

The broad isomer distributions of the primary/secondary diols in the wheat leaf wax suggest that they are also formed by a P450 hydroxylase. It seems plausible that the enzyme(s) converting alkanes into secondary alcohols (see above) can also hydroxylate primary alcohols into corresponding diols. Considering the clear predominance of C_28_ diol (compare Fig 9), very similar to the profile of precursor primary alcohols [30], it appears that the pool of alcohols is used non-discriminatively by the enzyme(s). Interestingly, both the secondary alcohols and diols had in-chain hydroxyls mainly on the methylene unit 12 carbons away from the methyl (or ω-) terminus, while the distance to the second methyl or alcohol terminus varied. We thus propose that the wheat MAH1-like enzyme may achieve its (limited) regio-specificity by tight binding of the short alkyl moiety of the alkane or primary alcohol substrates, effectively counting carbons in from the methyl terminus. It should also be noted that the C_28_ alcohol thus serving as substrate has an overall molecule geometry, including the carbon chain and the oxygen atom, resembling the C_29_ alkane homolog against which the enzyme(s) seemed to discriminate (compare Fig 9).

Primary/secondary diols with in-chain hydroxyl groups on several adjacent carbons, similar to those in wheat wax, had been reported for several other plant species before. For example, *Pisum sativum* leaves contain C_26_-C_28_ diols with 1,12- through 1,17-functionalities [15], while the eustigmatophyte *Nannochloropsis gaditana* has C_28_-C_36_ diols with 1,13- through 1,19-geometries [53]. Again it seems likely that the secondary hydroxyls of these diols may be introduced by P450 enzymes. In contrast, other diols were detected with the secondary hydroxyls exclusively on odd-numbered carbons (when counting from the primary OH), and are thought to be formed as elongation by-products. Examples for such compounds include the C_28_-C_38_ 1,5-diols in *Taxus baccata* needles [17], C_28_-C_32_ 1,11-diols in *Osmunda regalis* fronds [18], C_32_ 1,9-, 1,11- and 1,13-diols in *Myricaria germanica* leaves [13], or C_30_ 1,11-, C_32_ 1,13-, C_34_ 1,15- and C_36_ 1,17-diols in *Azolla filliculoides* whole plants [54].

Finally, esters of primary/secondary diols have scarcely been reported, including the C_46_-C_52_ esters of C_30_ 1,11-, C_32_ 1,13-, C_34_ 1,15- and C_36_ 1,17-diols of the fern *Azolla filliculoides* [54] and C_40_-C_52_ esters of C_30_ 1,5-, 1,7- and 1,9-diols of the moss *Funaria hygrometrica* [20]. It seems plausible that such esters are acylation products of corresponding free primary/secondary diols, formed by wax ester synthases. The diol esters found in wheat wax had nearly identical homolog and regiomer distributions as the accompanying free diols, suggesting that the responsible wax ester synthase shows no preference for the diol substrate. Instead, based on the chain length distribution of diol esters peaking at C_50_ (compare Fig 9A), it appears that the wax ester synthase shows high substrate preference for C_22_ acyl-CoA as its second substrate. Since the same preference was noted for a wax ester synthase forming unsubstituted VLC esters in the leaves of the same wheat cultivar [30], it is very likely that the same wax ester synthase produces esters of both primary alcohols and diols. This conclusion is in accordance with our finding that only the primary hydroxyl of the primary/secondary diols was esterified, but not the secondary group.

### Hydroxy-2-alkanol esters and oxo-2-alkanol esters in flag leaf and peduncle waxes

2-Alkanol esters have been identified in several grass species, typically as minor components associated with the much more prominent wax β-diketones [30,55,56]. Most previous analyses revealed only esters of 2-alkanols bearing no other functional groups, except for one report identifying 7-oxo-pentadecan-2-ol as a minor constituent of barley spike wax [45]. The same compound was thus now also detected in wheat waxes, together with its C_13_ homolog and 8-oxo isomers. Furthermore, we identified the four corresponding hydroxy-2-alkanols, with chain lengths and in-chain functional group positions matching those of the keto-2-alkanols, all esterified with various fatty acids.

The common overall chain length profiles and isomer distributions of the hydroxy-2-alkanol esters and oxo-2-alkanol esters identified here suggest a biosynthetic relationship between both compound classes. Considering that regiomers with functional groups on adjacent carbons were detected for all the 2-alkanol ester derivatives, it is likely that the in-chain functionalities are introduced by a cytochrome P450 hydroxylase similar to MAH1. Accordingly, we propose that wheat has a P450 enzyme catalyzing either a single hydroxylation leading to the hydroxy-2-alkanol esters or a double-hydroxylation to the corresponding oxo-2-alkanol esters.

The apparent regio-specificity of this enzyme, as a C-7/C-8 hydroxylase, clearly differs from that of the P450 discussed above for the formation of secondary alcohols and diols. Interestingly, both enzymes also appear to have different expression patterns, since the secondary alcohols/diols were found only in flag leaves, whereas the oxidized 2-alkanol esters were detected in both peduncle and flag leaf waxes (even though potential precursors for both product groups were likely present in both organs). Taken together, we conclude that *T*. *aestivum* cv. Bethlehem has at least two distinct P450 enzymes involved in wax biosynthesis, one being a C-12-specific hydroxylase forming secondary alcohols and diols, and the other one a C-7/8-specific hydroxylase involved in formation of oxidized 2-alkanol esters.

The matching chain length distributions of the esterified hydroxy/oxo-2-alkanols and 2-alkanols [30], both peaking at C_13/15_, indicate the latter could be the substrates for hydroxylation by this second hydroxylase targeting carbons C-7 and C-8 of the 2-alkanol moiety. The C-7 and C-8 positions found preferentially hydroxylated in 2-alkanols are very similar to those of hydroxyl groups in oxidized β-diketones (*e*.*g*., 8- and 9-hydroxy-hentriacontane-14,16-dione), relative to both ends of either the 2-alkanols or the alkyl moiety within the β-diketones [30]. It is thus plausible that the same hydroxylase is involved in the formation of hydroxy/oxo-2-alkanol esters and hydroxy-β-diketones. In fact, the geometry of the β-diketone, bearing a C_13_ alkyl tail on a -CO-CH_2_-CO- functionality, is fairly similar to that of the (C_15_) 2-alkanol esters, having a C_13_ alkyl tail on a–CHCH_3_-O-CO- functionality. The same P450 may thus accept either the β-diketone or the 2-alkanol ester as substrate for hydroxylation on C-7 or C-8. However, it cannot be ruled out that hydroxylation may occur in earlier stages of the pathways leading to 2-alkanol esters and β-diketones, rather than on the latter two products.

### 4-Alkylbutan-4-olides and internally branched alkanes in flag leaf and peduncle waxes

While δ-lactones have been identified as prominent components of the wax mixture on leaves of *Cerinthe minor* [47], the corresponding 4-alkylbutan-4-olides (4-alkyl-γ-lactones) have not been reported before as plant cuticular wax constituents. However, γ-lactones have been identified in several plant species, albeit without localizing them to a specific organ or tissue. For example, a homologous series of C_24_ to C_30_ γ-lactones was detected in the ground aerial parts of *Flourensia cernua* [57], the C_32_ γ-lactone in the aerial parts of *Pluchea lanceolata* [58], and an unsaturated C_21_ homolog in the stem bark of *Garcinia mannii* [59]. While the biosynthetic pathways leading to these structures remain unknown, it seems possible that they are formed via α-hydroxylation of acyl-CoA substrates similar to the reactions thought to lead to 1,2-bifunctional wax compounds [12]. The resulting α-hydroxyacyl-CoA intermediates might be elongated further by the FAE complex, leading to 4-hydroxyacyl-CoAs that, upon intramolecular esterification, would yield 4-alkylbutan-4-olides.

Finally, alkanes with an in-chain methyl branch had been described before as constituents of insect cuticular waxes [60], of wool wax [61], but also of plant cuticular waxes, namely leaf waxes of walnut tree [62] and spike waxes of barley [48]. It is important to note that, different from previous reports, the in-chain-branched alkane regiomers identified here in wheat wax had methyl groups separated by two carbons, located on odd-numbered carbons in the chain of homologs with odd chain lengths (even total carbon numbers) and on even-numbered carbons in even-numbered homologs (with odd total carbon numbers). Furthermore, odd-numbered chain lengths were more abundant than their even-numbered homologs, in the same way that odd-numbered *n*-alkanes are more abundant than those with even chain lengths. Based on these isomer patterns, we conclude that the methyl groups are most likely introduced during FAE-catalyzed elongation of acyl-CoA precursors, possibly by incorporating a methylmalonyl-CoA extender unit in lieu of the normal malonyl-CoA. The coverage of the branched alkanes (0.4 μg/cm^2^) was much lower than that of unbranched alkanes in the same wheat wax mixtures (1.5–4 μg/cm^2^) [30], indicating relatively low incorporation ratios of methylmalonate. Accordingly, alkanes with two or more methyl branches might be formed by the same mechanisms, however in very small quantities which could not be detected in the present study. Interestingly, barley spike waxes were reported to contain branched alkanes with very similar structures, but very different homolog and isomer distributions (characterized by similar abundance of compounds with methyl branches on adjacent carbons). This led to the conclusion that the barley branched alkanes are formed from unsaturated precursors via cyclopropane intermediates rather than by methylmalonyl-CoA incorporation [48].

## Conclusions

In this work, seven compound classes were identified and quantified in the wax mixtures covering flag leaf blades and peduncles of *T*. *aestivum* cv. Bethlehem. Three of them were secondary alcohols, primary/secondary diols and their esters with (very-) long chain acids, all of which were found as homologous series only in flag leaf wax. These three classes appeared biosynthetically related based on their similar secondary hydroxyl groups (on and around C-12 from the non-functionalized chain end). The hydroxy-2-alkanol esters and oxo-2-alkanol esters found in both organs are biosynthetically related to hydroxy-β-diketones, as suggested also by a common location of hydroxyl/oxo groups. We hypothesize that both compound groups, the secondary alcohols/diols and the oxidized 2-alkanol esters/β-diketones, are formed by two distinct P450 enzymes with C-12 and C-7/8 regio-specificity, respectively. In contrast, the two other compound classes identified here, 4-alkylbutan-4-olides and internally methyl-branched may be formed via α-oxidation and through incorporation of methylmalonyl-CoA into fatty acyl-CoA intermediates, respectively. Overall, we thus propose three specific variations from the normal wax biosynthesis pathways to occur in wheat, in the form of P450 oxidation, α-oxidation and methylmalonate incorporation.

## Supporting Information

S1 Table Characteristic fragments and relative abundances of secondary alcohols detected in wheat leaf wax. The fragments (*m/z*) of trimethylsilyl ether derivatives used to identify different secondary alcohol homologs and isomers are listed. Relative abundances (percent of respective homologs) were calculated from the abundances of the smaller isomer-specific fragments in a single, representative GC-MS run of the TLC fraction R_f_ 0.72 (fraction **A**).
(PDF)

S2 Table Characteristic fragments and relative abundances of diols with one primary and one secondary hydroxyl function detected in wheat leaf wax. The fragments (*m/z*) of bis(trimethylsilyl) ether derivatives used to identify different diol homologs and isomers are listed. Relative abundances (percent of respective homologs) were averages across the abundances of the smaller isomer-specific fragments in a single, representative GC-MS run of the TLC fraction R_f_ 0.30 (fraction **B**).
(PDF)

S3 Table Characteristic fragments of esterified diols with one primary and one secondary hydroxyl function detected in wheat leaf wax. The fragments (*m/z*) of trimethylsilyl ether derivatives used to identify different diol ester homologs and isomers are listed (fraction **C**).
(PDF)

S4 Table Characteristic fragments of hydroxy-2-alkanol esters detected in wheat leaf wax. The fragments (*m/z*) of trimethylsilyl ether derivatives used to identify different ester homologs and isomers are listed (fraction **D**).
(PDF)

S5 Table Characteristic fragments of oxo-2-alkanol esters detected in wheat leaf wax. The fragments (*m/z*) used to identify different ester homologs and isomers are listed (fraction **E**).
(PDF)

S6 Table Characteristic fragments of 4-alkylbutan-4-olides detected in wheat leaf wax. The fragments (*m/z*) used to identify different homologs and isomers are listed (fraction **F**).
(PDF)

S7 Table Characteristic fragments and relative abundances of branched alkanes detected in wheat leaf wax. The fragments (*m/z*) of used to identify different secondary alkane homologs and isomers are listed. Relative abundances (percent of respective homologs) were calculated from the abundances of the even-numbered, heavier isomer-specific fragments in a single, representative GC-MS run of the TLC fraction R_f_ 1.00 (fraction **G**).
(PDF)

## Abbreviations

BSTFA: *N*,*O*-bis(trimethylsilyl)trifluoroacetamide
D: diameter
ER: endoplasmic reticulum
FAE: fatty acid elongase
FAS: fatty acid synthase
FID: flame ionization detector
GC: gas chromatography
L: length
LC: long-chain
MAH1: midchain alkane hydroxylase 1
MS: mass spectrometry
PDMS: polydimethylsiloxane
TLC: thin layer chromatography
VLC: very-long-chain.
