# Supplementary material for: Identification of In-Chain-Functionalized Compounds and Methyl-Branched Alkanes in Cuticular Waxes of Triticum aestivum cv. Bethlehem
Source: PLoS One. 2016 Nov 7;11(11):e0165827. doi: 10.1371/journal.pone.0165827 (PMC5098774; doi:10.1371/journal.pone.0165827)
Supplement: S1 Table — The fragments (m/z) of trimethylsilyl ether derivatives used to identify different secondary alcohol homologs and isomers are listed. Relative abundances (percent of respective homologs) were calculated from the abundances of the smaller isomer-specific fragments in a single, representative GC-MS run of the TLC fraction Rf 0.72 (fraction A). (PDF) [file pone.0165827.s001.pdf]

S1 Table. Characteristic fragments and relative abundances of secondary alcohols detected in wheat leaf wax. The fragments (*m/z*) of trimethylsilyl ether derivatives used to identify different secondary alcohol homologs and isomers are listed. Relative abundances (percent of respective homologs) were calculated from the abundances of the smaller isomer-specific fragments in a single, representative GC-MS run of the TLC fraction R_f 0.72 (fraction **A**).

**Compound**	**Fragments characteristic of homolog (*m/z*)**		**Fragments characteristic of isomer (*m/z*)**		**Relative isomer abundance (% of homolog)**
Pentacosan-8-ol	350	425	201	341	17
Pentacosan-9-ol			215	327	n.q.
Pentacosan-10-ol			229	313	25
Pentacosan-11-ol			243	299	n.q.
Pentacosan-12-ol			257	285	58
Pentacosan-13-ol			271		n.q.
Heptacosan-9-ol	378	453	215	355	2
Heptacosan-10-ol			229	341	8
Heptacosan-11-ol			243	327	24
Heptacosan-12-ol			257	313	32
Heptacosan-13-ol			271	299	18
Heptacosan-14-ol			285		15
Nonacosan-9-ol	406	481	215	383	1
Nonacosan-10-ol			229	369	8
Nonacosan-11-ol			243	355	35
Nonacosan-12-ol			257	341	28
Nonacosan-13-ol			271	327	14
Nonacosan-14-ol			285	313	7
Nonacosan-15-ol			299		7
Hentriacontan-8-ol	434	509	201	425	1
Hentriacontan-9-ol			215	411	2
Hentriacontan-10-ol			229	397	5
Hentriacontan-11-ol			243	383	16
Hentriacontan-12-ol			257	369	61
Hentriacontan-13-ol			271	355	10
Hentriacontan-14-ol			285	341	2
Hentriacontan-15-ol			299	327	1
Hentriacontan-16-ol			313		1
Tritriacontan-9-ol	462	537	215	439	1
Tritriacontan-10-ol			229	425	3
Tritriacontan-11-ol			243	411	14
Tritriacontan-12-ol			257	397	65
Tritriacontan-13-ol			271	383	12
Tritriacontan-14-ol			285	369	4
Tritriacontan-15-ol			299	355	1
Tritriacontan-16-ol			313	341	tr
Tritriacontan-17-ol			327		1
Pentratriacontan-8-ol	490	565	201	481	1
Pentatriacontan-9-ol			215	467	4
Pentatriacontan-10-ol			229	453	13
Pentatriacontan-11-ol			243	439	36
Pentatriacontan-12-ol			257	425	39
Pentatriacontan-13-ol			271	411	6
Pentatriacontan-14-ol			285	397	tr
Pentatriacontan-15-ol			299	383	tr
Pentatriacontan-16-ol			313	369	tr
Pentatriacontan-17-ol			327	355	1
Pentatriacontan-18-ol			341		1
