# Supplementary material for: Identification of In-Chain-Functionalized Compounds and Methyl-Branched Alkanes in Cuticular Waxes of Triticum aestivum cv. Bethlehem
Source: PLoS One. 2016 Nov 7;11(11):e0165827. doi: 10.1371/journal.pone.0165827 (PMC5098774; doi:10.1371/journal.pone.0165827)
Supplement: S2 Table — The fragments (m/z) of bis(trimethylsilyl) ether derivatives used to identify different diol homologs and isomers are listed. Relative abundances (percent of respective homologs) were averages across the abundances of the smaller isomer-specific fragments in a single, representative GC-MS run of the TLC fraction Rf 0.30 (fraction B). (PDF) [file pone.0165827.s002.pdf]

S2 Table. Characteristic fragments and relative abundances of diols with one primary and one secondary hydroxyl function detected in wheat leaf wax. The fragments (*m/z*) of bis(trimethylsilyl) ether derivatives used to identify different diol homologs and isomers are listed. Relative abundances (percent of respective homologs) were averages across the abundances of the smaller isomer-specific fragments in a single, representative GC-MS run of the TLC fraction R_f 0.30 (fraction **B**).

**Compound**	**Fragments characteristic of homolog (*m/z*)**			**Fragments characteristic of isomer (*m/z*)**			**Relative isomer abundance (% of homolog)**
Hexacosane-1,19-diol	437	452	527	201	443	353	4
Hexacosane-1,18-diol				215	429	339	2
Hexacosane-1,17-diol				229	415	325	5
Hexacosane-1,16-diol				243	401	311	33
Hexacosane-1,15-diol				257	387	297	52
Hexacosane-1,14-diol				271	373	283	4
Hexacosane-1,13-diol				285	359	269	tr
Octacosane-1,21-diol	465	480	555	201	471	381	1
Octacosane-1,20-diol				215	457	367	3
Octacosane -1,19-diol				229	443	353	9
Octacosane -1,18-diol				243	429	339	23
Octacosane -1,17-diol				257	415	325	30
Octacosane -1,16-diol				271	401	311	25
Octacosane -1,15-diol				285	387	297	9
Octacosane -1,14-diol				299	373	283	tr
Triacontane-1,23-diol	493	508	583	201	499	409	3
Triacontane-1,22-diol				215	485	395	3
Triacontane-1,21-diol				229	471	381	8
Triacontane-1,20-diol				243	457	367	30
Triacontane-1,19-diol				257	443	353	40
Triacontane-1,18-diol				271	429	339	8
Triacontane-1,17-diol				285	415	325	2
Triacontane-1,16-diol				299	401	311	1
