# Supplementary material for: Identification of In-Chain-Functionalized Compounds and Methyl-Branched Alkanes in Cuticular Waxes of Triticum aestivum cv. Bethlehem
Source: PLoS One. 2016 Nov 7;11(11):e0165827. doi: 10.1371/journal.pone.0165827 (PMC5098774; doi:10.1371/journal.pone.0165827)
Supplement: S3 Table — The fragments (m/z) of trimethylsilyl ether derivatives used to identify different diol ester homologs and isomers are listed (fraction C). (PDF) [file pone.0165827.s003.pdf]

S3 Table. Characteristic fragments of esterified diols with one primary and one secondary hydroxyl function detected in wheat leaf wax. The fragments (*m/z*) of trimethylsilyl ether derivatives used to identify different diol ester homologs and isomers are listed (fraction **C**).

**Compound**	**Fragments characteristic of homolog (*m/z*)**	**Fragments characteristic of isomer (*m/z*)**	
Octacosane-1,20-diol palmitate	239 257 313 329	215	623
Octacosane-1,19-diol palmitate		229	609
Octacosane-1,18-diol palmitate		243	595
Octacosane-1,17-diol palmitate		257	581
Octacosane-1,16-diol palmitate		271	567
Octacosane-1,15-diol palmitate		285	553
Octacosane-1,14-diol palmitate		299	539
Octacosane-1,20-diol stearate	267 285 341 357	215	651
Octacosane-1,19-diol stearate		229	637
Octacosane-1,18-diol stearate		243	623
Octacosane-1,17-diol stearate		257	609
Octacosane-1,16-diol stearate		271	595
Octacosane-1,15-diol stearate		285	581
Octacosane-1,14-diol stearate		299	567
Octacosane-1,20-diol arachidate	295 313 369 385	215	679
Octacosane-1,19-diol arachidate		229	665
Octacosane-1,18-diol arachidate		243	651
Octacosane-1,17-diol arachidate		257	637
Octacosane-1,16-diol arachidate		271	623
Octacosane-1,15-diol arachidate		285	609
Octacosane-1,14-diol arachidate		299	595
Octacosane-1,20-diol behenate	323 341 397 413	215	707
Octacosane-1,19-diol behenate		229	693
Octacosane-1,18-diol behenate		243	679
Octacosane-1,17-diol behenate		257	665
Octacosane-1,16-diol behenate		271	651
Octacosane-1,15-diol behenate		285	637
Octacosane-1,14-diol behenate		299	623
Octacosane-1,20-diol lignocerate	351 369 425 441	215	735
Octacosane-1,19-diol lignocerate		229	721
Octacosane-1,18-diol lignocerate		243	707
Octacosane-1,17-diol lignocerate		257	693
Octacosane-1,16-diol lignocerate		271	679
Octacosane-1,15-diol lignocerate		285	665
Octacosane-1,14-diol lignocerate		299	651
