# Supplementary material for: Identification of In-Chain-Functionalized Compounds and Methyl-Branched Alkanes in Cuticular Waxes of Triticum aestivum cv. Bethlehem
Source: PLoS One. 2016 Nov 7;11(11):e0165827. doi: 10.1371/journal.pone.0165827 (PMC5098774; doi:10.1371/journal.pone.0165827)
Supplement: S4 Table — The fragments (m/z) of trimethylsilyl ether derivatives used to identify different ester homologs and isomers are listed (fraction D). (PDF) [file pone.0165827.s004.pdf]

S4 Table. Characteristic fragments of hydroxy-2-alkanol esters detected in wheat leaf wax. The fragments (*m/z*) of trimethylsilyl ether derivatives used to identify different ester homologs and isomers are listed (fraction **D**).

**Compound**	**Alternative name**	**Fragments characteristic of homolog (*m/z*)**	**Fragments characteristic of isomer type: regiomer (*m/z*)**	**metamer (*m/z*)**
Tridecane-2,8-diol stearate	8-Hydroxytridecan-2-ol stearate	539	173 483 199 109	*267 285 341 357*
Tridecane-2,7-diol stearate	7-Hydroxytridecan-2-ol stearate		187 469 185 95	
Pentadecane-2,8-diol palmitate	8-Hydroxypentadecan-2-ol palmitate		201 455 199 109	*239 257 313 329*
Pentadecane-2,7-diol palmitate	7-Hydroxypentadecan-2-ol palmitate		215 441 185 95	
Tridecane-2,8-diol arachidate	8-Hydroxytridecan-2-ol arachidate	567	173 511 199 109	*295 312 369 385*
Tridecane-2,7-diol arachidate	7-Hydroxytridecan-2-ol arachidate		187 497 185 95	
Pentadecane-2,8-diol stearate	8-Hydroxypentadecan-2-ol stearate		201 483 199 109	*267 285 341 357*
Pentadecane-2,7-diol stearate	7-Hydroxypentadecan-2-ol stearate		215 469 185 95	
Tridecane-2,8-diol behenate	8-Hydroxytridecan-2-ol behenate	595	173 539 199 109	*323 340 397 413*
Tridecane-2,7-diol behenate	7-Hydroxytridecan-2-ol behenate		187 525 185 95	
Pentadecane-2,8-diol arachidate	8-Hydroxypentadecan-2-ol arachidate		201 511 199 109	*295 312 369 385*
Pentadecane-2,7-diol arachidate	7-Hydroxypentadecan-2-ol arachidate		215 497 185 95	
Tridecane-2,8-diol lignocerate	8-Hydroxytridecan-2-ol lignocerate	623	173 567 199 109	*351 368 425 441*
Tridecane-2,7-diol lignocerate	7-Hydroxytridecan-2-ol lignocerate		187 553 185 95	
Pentadecane-2,8-diol behenate	8-Hydroxypentadecan-2-ol behenate		201 539 199 109	*323 340 397 413*
Pentadecane-2,7-diol behenate	7-Hydroxypentadecan-2-ol behenate		215 525 185 95	
Tridecane-2,8-diol cerotate	8-Hydroxytridecan-2-ol cerotate	651	173 595 199 109	*379 396 453 469*
Tridecane-2,7-diol cerotate	7-Hydroxytridecan-2-ol cerotate		187 581 185 95	
Pentadecane-2,8-diol lignocerate	8-Hydroxypentadecan-2-ol lignocerate		201 567 199 109	*351 368 425 441*
Pentadecane-2,7-diol lignocerate	7-Hydroxypentadecan-2-ol lignocerate		215 553 185 95	
