# Supplementary material for: Identification of In-Chain-Functionalized Compounds and Methyl-Branched Alkanes in Cuticular Waxes of Triticum aestivum cv. Bethlehem
Source: PLoS One. 2016 Nov 7;11(11):e0165827. doi: 10.1371/journal.pone.0165827 (PMC5098774; doi:10.1371/journal.pone.0165827)
Supplement: S5 Table — The fragments (m/z) used to identify different ester homologs and isomers are listed (fraction E). (PDF) [file pone.0165827.s005.pdf]

S5 Table. Characteristic fragments of oxo-2-alkanol esters detected in wheat leaf wax. The fragments (*m/z*) used to identify different ester homologs and isomers are listed (fraction **E**).

**Compound**	**Alternative name**	**Fragments characteristic of homolog (*m/z*)**	**Fragments characteristic of isomer type: regiomer (*m/z*)**	**metamer (*m/z*)**
2-Hydroxytridecan-8-one stearate	8-oxotridecan-2-ol stearate	-	99 114 424	*197 267 285*
2-Hydroxytridecan-7-one stearate	7-oxotridecan-2-ol stearate		113 128 410	
2-Hydroxypentadecan-8-one palmitate	8-oxopentadecan-2-ol palmitate		127 142 396	*225 239 257*
2-Hydroxypentadecan-7-one palmitate	7-oxopentadecan-2-ol palmitate		141 156 382	
2-Hydroxytridecan-8-one arachidate	8-oxotridecan-2-ol arachidate	-	99 114 452	*197 295 313*
2-Hydroxytridecan-7-one arachidate	7-oxotridecan-2-ol arachidate		113 128 438	
2-Hydroxypentadecan-8-one stearate	8-oxopentadecan-2-ol stearate		127 142 424	*225 267 285*
2-Hydroxypentadecan-7-one stearate	7-oxopentadecan-2-ol stearate		141 156 410	
2-Hydroxytridecan-8-one behenate	8-oxotridecan-2-ol behenate	-	99 114 480	*197 323 341*
2-Hydroxytridecan-7-one behenate	7-oxotridecan-2-ol behenate		113 128 466	
2-Hydroxypentadecan-8-one arachidate	8-oxopentadecan-2-ol arachidate		127 142 452	*225 295 313*
2-Hydroxypentadecan-7-one arachidate	7-oxopentadecan-2-ol arachidate		141 156 438	
2-Hydroxytridecan-8-one lignocerate	8-oxotridecan-2-ol lignocerate	-	99 114 508	*197 351 369*
2-Hydroxytridecan-7-one lignocerate	7-oxotridecan-2-ol lignocerate		113 128 494	
2-Hydroxypentadecan-8-one behenate	8-oxopentadecan-2-ol behenate		127 142 480	*225 323 341*
2-Hydroxypentadecan-7-one behenate	7-oxopentadecan-2-ol behenate		141 156 466	
2-Hydroxytridecan-8-one cerotate	8-oxotridecan-2-ol cerotate	-	99 114 536	*197 379 397*
2-Hydroxytridecan-7-one cerotate	7-oxotridecan-2-ol cerotate		113 128 522	
2-Hydroxypentadecan-8-one lignocerate	8-oxopentadecan-2-ol lignocerate		127 142 508	*225 351 369*
2-Hydroxypentadecan-7-one lignocerate	7-oxopentadecan-2-ol lignocerate		141 156 494	
