# Supplementary material for: Identification of In-Chain-Functionalized Compounds and Methyl-Branched Alkanes in Cuticular Waxes of Triticum aestivum cv. Bethlehem
Source: PLoS One. 2016 Nov 7;11(11):e0165827. doi: 10.1371/journal.pone.0165827 (PMC5098774; doi:10.1371/journal.pone.0165827)
Supplement: S6 Table — The fragments (m/z) used to identify different homologs and isomers are listed (fraction F). (PDF) [file pone.0165827.s006.pdf]

S6 Table. Characteristic fragments of 4-alkylbutan-4-olides detected in wheat leaf wax. The fragments (*m/z*) used to identify different homologs and isomers are listed (fraction **F**).

**Compound**	**Alternative name**	**Fragments characteristic of homolog (*m/z*)**			
4-Docosylbutan-4-olide	4-Hydroxyhexacosanoic acid γ-lactone	332	358	376	394
4-Tetracosylbutan-4-olide	4-Hydroxyoctacosanoic acid γ-lactone	360	386	404	422
4-Hexacosylbutan-4-olide	4-Hydroxytriacontanoic acid γ-lactone	388	414	432	450
4-Octacosylbutan-4-olide	4-Hydroxydotriacontanoic acid γ-lactone	416	442	460	478
