# Supplementary material for: Identification of In-Chain-Functionalized Compounds and Methyl-Branched Alkanes in Cuticular Waxes of Triticum aestivum cv. Bethlehem
Source: PLoS One. 2016 Nov 7;11(11):e0165827. doi: 10.1371/journal.pone.0165827 (PMC5098774; doi:10.1371/journal.pone.0165827)
Supplement: S7 Table — The fragments (m/z) of used to identify different secondary alkane homologs and isomers are listed. Relative abundances (percent of respective homologs) were calculated from the abundances of the even-numbered, heavier isomer-specific fragments in a single, representative GC-MS run of the TLC fraction Rf 1.00 (fraction G). (PDF) [file pone.0165827.s007.pdf]

S7 Table. Characteristic fragments and relative abundances of branched alkanes detected in wheat leaf wax. The fragments (*m/z*) of used to identify different secondary alkane homologs and isomers are listed. Relative abundances (percent of respective homologs) were calculated from the abundances of the even-numbered, heavier isomer-specific fragments in a single, representative GC-MS run of the TLC fraction R_f 1.00 (fraction **G**).

**Compound**	**Chain length**	**Carbon number**	**Fragments characteristic of homolog (*m/z*)**		**Fragments characteristic of isomer (*m/z*)**		**Relative isomer abundance (% of homolog)**
9-Methylheptacosane	27	28	379	394	140/141	280/281	21
10-Methylheptacosane	27	28			154/155	266/267	7
11-Methylheptacosane	27	28			168/169	252/253	47
12-Methylheptacosane	27	28			182/183	238/239	1
13-Methylheptacosane	27	28			196/197	224/225	25
14-Methylheptacosane	27	28			210/211		tr
9-Methyloctacosane	28	29	393	408	140/141	294/295	16
10-Methyloctacosane	28	29			154/155	280/281	28
11-Methyloctacosane	28	29			168/169	266/267	10
12-Methyloctacosane	28	29			182/183	252/253	46
13-Methyloctacosane	28	29			196/197	238/239	tr
14-Methyloctacosane	28	29			210/211	224/225	1
9-Methylnonacosane	29	30	407	422	140/141	308/309	15
10-Methylnonacosane	29	30			154/155	294/295	5
11-Methylnonacosane	29	30			168/169	280/281	45
12-Methylnonacosane	29	30			182/183	266/267	1
13-Methylnonacosane	29	30			196/197	252/253	25
14-Methylnonacosane	29	30			210/211	238/239	2
15-Methylnonacosane	29	30			224/225		7
9-Methyltriacontane	30	31	421	436	140/141	322/323	10
10-Methyltriacontane	30	31			154/155	308/309	29
11-Methyltriacontane	30	31			166/167	294/295	9
12-Methyltriacontane	30	31			182/183	280/281	39
13-Methyltriacontane	30	31			196/197	266/267	6
14-Methyltriacontane	30	31			210/211	252/253	6
15-Methyltriacontane	30	31			224/225	238/239	1
9-Methylhentriacontane	31	32	435	450	140/141	336/337	16
10-Methylhentriacontane	31	32			154/155	322/323	5
11-Methylhentriacontane	31	32			168/169	308/309	49
12-Methylhentriacontane	31	32			182/183	294/295	4
13-Methylhentriacontane	31	32			196/197	280/281	18
14-Methylhentriacontane	31	32			210/211	266/267	3
15-Methylhentriacontane	31	32			224/225	252/253	5
16-Methylhentriacontane	31	32			238/239		1
9-Methyldotriacontane	32	33	449	464	140/141	350/351	13
10-Methyldotriacontane	32	33			154/155	336/337	27
11-Methyldotriacontane	32	33			168/169	322/323	8
12-Methyldotriacontane	32	33			182/183	308/309	43
13-Methyldotriacontane	32	33			196/197	294/295	4
14-Methyldotriacontane	32	33			210/211	280/281	4
15-Methyldotriacontane	32	33			224/225	266/267	1
16-Methyldotriacontane	32	33			238/239	252/253	1
